# Expression Analyses of Genes Related to Multixenobiotic Resistance in *Mytilus galloprovincialis* after Exposure to Okadaic Acid-Producing *Dinophysis acuminata*

**DOI:** 10.3390/toxins13090614

**Published:** 2021-09-01

**Authors:** Roi Martínez-Escauriaza, Vanessa Lozano, M. Luz Pérez-Parallé, Juan Blanco, José L. Sánchez, Antonio J. Pazos

**Affiliations:** 1 Departamento de Bioquímica y Biología Molecular, Instituto de Acuicultura, Universidade de Santiago de Compostela, 15782 Santiago de Compostela, Spain; roimartinez@hotmail.com (R.M.-E.); palova_marina@hotmail.com (V.L.); joseluis.sanchez@usc.es (J.L.S.); antonioj.pazos@usc.es (A.J.P.); 2 Centro de Investigacións Mariñas, Xunta de Galicia, Pedras de Corón s/n Apdo. 13, 36620 Vilanova de Arousa, Spain; juan.carlos.blanco.perez@xunta.gal

**Keywords:** P-glycoprotein, *mdr*, *mrp*, ABC transporters, DSP toxins, MXR

## Abstract

The mussel *Mytilus galloprovincialis* is one of the most important aquaculture species in Europe. Its main production problem is the accumulation of toxins during coastal blooms, which prevents mussel commercialization. P-glycoprotein (ABCB1/MDR1/P-gp) is part of the multixenobiotic resistance system in aquatic organisms, and okadaic acid, the main DSP toxin, is probably a substrate of the P-gp-mediated efflux. In this study, the presence and possible role of P-gp in the okadaic acid detoxification process was studied in *M. galloprovincialis*. We identified, cloned, and characterized two complete cDNAs of *mdr1* and *mdr2* genes. MgMDR1 and MgMDR2 predicted proteins had the structure organization of ABCB full transporters, and were identified as P-gp/MDR/ABCB proteins. Furthermore, the expression of *mdr* genes was monitored in gills, digestive gland, and mantle during a cycle of accumulation-elimination of okadaic acid. *Mdr1* significantly increased its expression in the digestive gland and gills, supporting the idea of an important role of the MDR1 protein in okadaic acid efflux out of cells in these tissues. The expression of *M. galloprovincialis* *mrp2*, a multidrug associated protein (MRP/ABCC), was also monitored. As in the case of *mdr1*, there was a significant induction in the expression of *mrp2* in the digestive gland, as the content of okadaic acid increased. Thus, P-gp and MRP might constitute a functional defense network against xenobiotics, and might be involved in the resistance mechanisms to DSP toxins.

## 1. Introduction

One of the most critical problems in bivalve aquaculture around the world is the accumulation of phycotoxins produced by harmful microalgae during coastal blooms. The microalgae that produce and contain the toxins are ingested by the bivalve mollusks that accumulate, transform, and eliminate these toxins. Any knowledge regarding these processes is of the greatest importance, in order to predict the course of a toxic bloom, and, therefore, to minimize its consequences [1]. There is a great variety of different phycotoxins among which several stand out, such as paralytic shellfish poisoning (PSP) toxins, amnesic shellfish poisoning (ASP) toxins, neurotoxic shellfish poisoning (NSP) toxins, diarrhetic shellfish poisoning (DSP) toxins, and azaspiracid shellfish poisoning (AZP) toxins [2,3]. Of these, DSP toxins, commonly associated with some microalgae of the genus *Dinophysis* and *Prorocentrum* cause a serious syndrome that produces severe gastrointestinal disorders in humans. DSP toxins are polyether molecules of a lipophilic nature; included in this group are okadaic acid (OA) and the structurally related dinophysistoxin-1 (DTX1) and dinophysistoxin-2 (DTX2), as well as several derivative forms [2]. It has been reported that OA inhibits the activity of protein threonine/serine phosphatase types 1(PP1) and 2 (PP2A) in yeasts, higher plants, and mammals. Thus, OA blocks the dephosphorylation of proteins that are substrates of protein kinases, affecting many basic processes, such as the regulation of gene expression, cell-cycle control, cell adhesion, apoptosis, or cytoskeleton dynamics [4,5]. It has also been demonstrated that low concentrations of OA have cytotoxic and mutagenic effects on different cell lines and on different bivalve tissues [6,7,8].

In addition to phycotoxins, anthropogenic pollutants and other natural toxins are present in the aquatic environment and pose serious threats to the development and production of aquatic organisms. In spite of these effects, bivalves and many other aquatic species are able to grow normally and survive in such conditions [9]. This ability was named the multixenobiotic resistance mechanism (MXR) by Kurelec [10], and it is similar to the multidrug resistance (MDR) observed in lines of tumor cells that are resistant to anticancer drugs. The MXR is a defense mechanism against environmental pollution that pumps various xenobiotics out of the cell, thus preventing their accumulation and toxic effects [11,12,13,14]. The MXR mechanism is mediated through several membrane transporters belonging to the ATP-binding cassette (ABC) family. Among the ABC proteins, members of the multidrug resistance associated protein (ABCC/MRP), the breast cancer resistance protein (ABCG2/BCRP), and the P-glycoprotein (P-gp, ABCB/MDR) are toxicologically relevant [15,16,17].

Several authors have revealed the presence of P-gp in bivalves and its important role in the detoxification of heavy metals and organic chemicals [18,19,20,21]; however, few studies have assessed the role of P-gp in the resistance mechanisms against poisoning by DSP toxins [7,22,23,24]. P-glycoprotein is a good candidate to expel okadaic acid out of the cells in bivalve mollusks because it transports a wide variety of structurally unrelated hydrophobic amphipathic compounds (such as okadaic acid) across membranes [25]. Furthermore, both functional and biochemical data support the proposal that okadaic acid is a substrate of the P-gp-mediated efflux activity in rat pituitary GH3 cells [6]. In addition, Ehlers et al. [26] have shown that OA is a substrate of human P-gp and that P-gp is involved in the elimination of OA from cells using two different transwell models: (i) caco-2 cell monolayer endogenously expressing human P-gp, simulating the intestinal barrier and (ii) MDCK-II cell monolayer stably over-expressing P-gp.

In this study, the Mediterranean mussel (*Mytilus galloprovincialis* Lamarck, 1819) was selected as a model organism to study the presence and possible role of P-gp in the okadaic acid detoxification process. *M. galloprovincialis* is one of the most important aquaculture species in Europe. Its production is concentrated mainly in Galicia (NW Spain), with an average yield of about 200,000 tons per year, and it is extremely important not only economically but also socially. Mussel aquaculture in Galicia (Spain) has not suffered biological or health problems, unlike other areas of Europe [27]. However, the presence of more frequent toxic episodes of phytoplanktonic origin is threatening the production of mussels and other marine bivalves in this area, and preventing its commercialization for long periods [28]. It has been suggested that one of the ways to reduce the impact caused by these episodes could be achieved through selective breeding programs, with strains of mussels that have a lower toxin uptake and/or a better detoxification [29]. A more in depth understanding of the metabolism of okadaic acid and the mechanisms of elimination in mussel tissues will bring about new insights into these detoxification systems, and will offer extremely valuable tools to aid the design of the breeding programs. As indicated above, P-gp is a priori an ideal candidate to transport okadaic acid out of mussel cells. However, to date, little is known about P-gp proteins and its genes in *M. galloprovincialis*, except for a short fragment of 447 bp that is very well conserved not only among P-gp proteins but also in the ABC protein family [30]. This short fragment is unsuitable for gene expression studies. It is necessary to have complete sequences, to avoid the presence of pseudogenes and to identify different splicing products. In the present study, we have identified, cloned, and characterized two complete cDNA sequences of P-gp (*Mgmdr1* and *Mgmdr2*) from *M. galloprovincialis*, in order to provide insights into the role of P-gp in okadaic acid detoxification. The mussel accumulation of okadaic acid and the expression patterns of *Mgmdr1* and *Mgmdr2* in the digestive gland, gills, and mantle were studied in *M. galloprovincialis* that were naturally contaminated with okadaic acid from a bloom of *Dinophysis acuminata* (Claparede and Lachmann, 1859). A possible role of these genes in detoxification processes is discussed.

## 2. Results

### 2.1. Cloning and Characterization of mdr Genes

**Figure 1:**
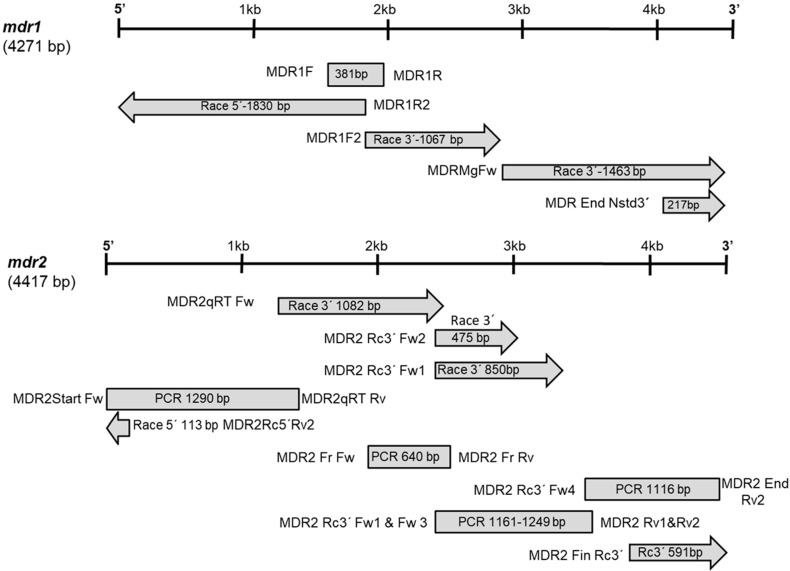
Cloning strategy to obtain the *M. galloprovincialis mdr* 1 and *mdr2* genes. Boxes and arrows indicate the relative position of the fragments obtained by PCR amplification or RACE, respectively.

Two full-length transcripts coding for ABCB (MDR) transporters from *M. galloprovincialis* were obtained by RT-PCR and RACE techniques (Figure 1). 

**Figure 2:**
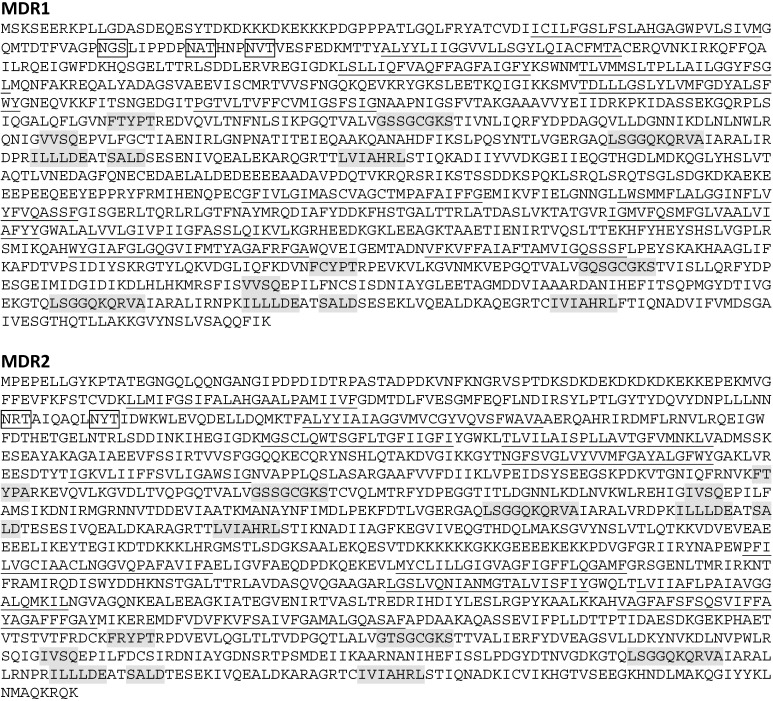
Deduced amino acid sequences of MDR1 and MDR2 from *M. galloprovincialis*. Underlined sections represent MDR1 and MDR2 predicting the structural organization of ABCB full transporters in two halves, each with a transmembrane domain (TMD) and a nucleotide binding domain (NBD), containing the characteristic and highly conserved motifs of ABC transporters: the A-loop, Walker A, Q-loop, Walker B, D-loop, and H-loop. The ABC transporter family signature (C motif), specifically the one that identifies the ABCB subfamily LSGGQKQRVA, is present (Figure 2 and Figure 3). Each TMD presented six transmembrane helices. MDR1 contained three *N*-glycosylation sequons at positions Asn^91^-Gly^92^-Ser^93^, Asn^100^-Ala^101^-Thr^102^, and Asn^106^-Val^107^-Thr^108^. MDR2 only presented two *N*-glycosylation sequons at positions Asn^161^-Arg^162^-Thr^163^ and Asn^170^-Tyr^171^-Thr^172^. In both cases they are located in the extracellular loop at NH_2_-terminal end of TMD1 (Figure 3).

The cDNAs finally obtained were 4271bp for *mdr1* and 4417 bp for *mdr2* (accession numbers FM999809 and HF912273, respectively). Open reading frames (ORF) encode 1307 amino acid residues with a predicted molecular mass of 143.91 kDa for *mdr1* and 1367 amino acid residues with a molecular mass of 150.37 kDa for *mdr2* (Figure 2). The *mdr1* sequence included a 189 bp 5′-untranslated region (UTR) and a 161 bp 3′-UTR. The *mdr2* sequence included a 117 bp 5′-UTR and a 196 bp 3′-UTR (Table 1).

The deduced amino acid sequence of the *M. galloprovincialis mdr1* and *mdr2* reveals considerable similarity with the MDR from bivalves and other organisms, confirming their identities as MDR. MgMDR1 and MgMDR2 showed considerable similarity with other ABCB proteins. After a homology search among bivalves, MgMDR1 showed 95% identity with *Mytilus californianus* ABCB1 (ABS83556.1); 92% identity with *Mytilus coruscus* ABCB1 (CAC5356955.1); 64% identity with *Crassostrea virginica* Mrp1 (XP022339240.1); 63% identity with *Crassostrea gigas* ABCB1 (XP011448242.2), *Anadara sativa* (AID66618.1), and *Tegillarca granosa* (AID66619.1) P-glycoproteins; and 62% identity with *Mizuhopecten yessoensis* Mrp1 (XP021368379.1). Furthermore, MgMDR1 showed 48% identity with ABCB1 and ABCB4 from *Homo sapiens* (P08183 and P21439, respectively). MgMDR2 showed 97% identity with *Mytilus coruscus* ABCB (QDF46975.1); 62% identity with *Pecten maximus* ABCB (XP033762432.1), *Crassostrea angulata* ABCB (ALF36867.1), and *Crassostrea virginica* Mrp1 (XP022303216.1); 61% with *Azumapecten farreri* p-glycoprotein (ACL80139.3); 60% identity with *Ruditapes philippinarum* P-glycoprotein (AID66617.1); 55% identity with *H. sapiens* ABCB1 and ABCB4 (P08183 and P21439, respectively); and 51% identity with *Mytilus galloprovincialis* MDR1.

**Figure 3:**
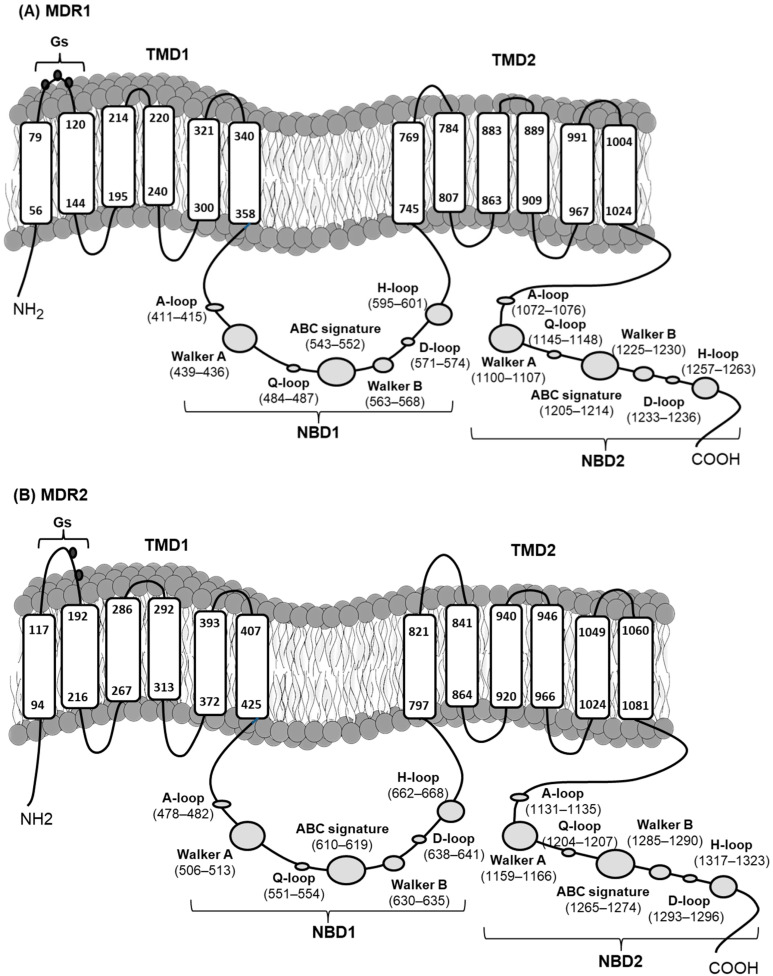
Topologies of *M. galloprovincialis* MDR1 (**A**) and MDR2 (**B**) proteins, showing the transmembrane domains (TMD), containing 6 membrane-spanning α-helices, and the nucleotide binding domains (NBD) that contained the typical and highly conserved motifs of ABC transporters. Putative *N*-glycosylation sites (Gs) are also marked.

**Figure 4:**
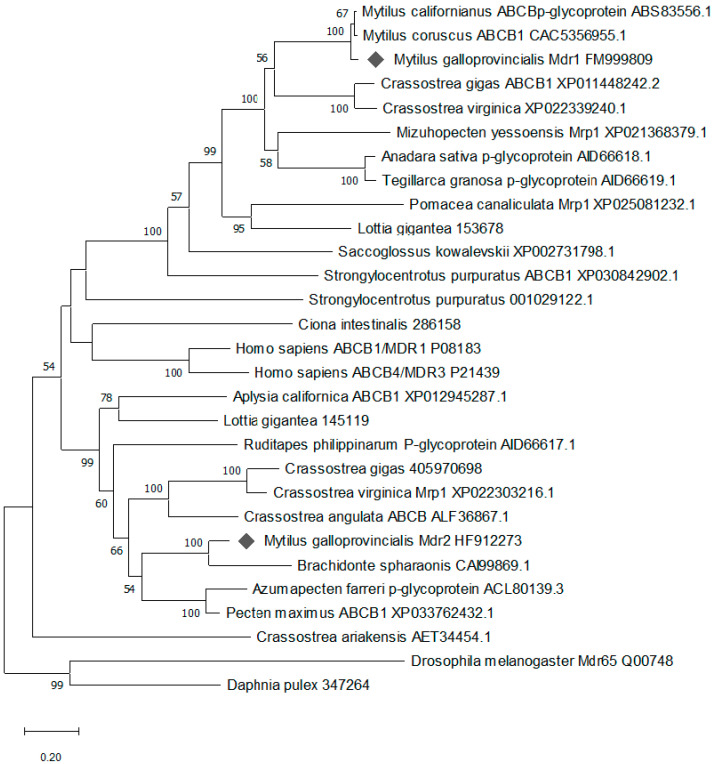
Phylogenetic analysis of MDR1 and MDR2 sequences from *M. galloprovincialis*. Multiple alignments of the selected sequences were conducted by Clustal W2, and phylogenetic analysis by the maximum likelihood method was conducted in MEGA X. Numbers on the branches indicate bootstrap support values. The scale shows the number of amino acid substitutions per site. MgMDR1 and MgMDR2 are marked by grey diamonds. Accession numbers of sequences used in alignments are shown.

Phylogenetic analysis by the maximum likelihood method supported the assignment of MgMDR1 and MgMDR2 as ABCB transporters (Figure 4). MgMDR1 was assigned to a cluster with orthologues from other bivalves *M. californianus*, *M. coruscus*, *M. yessoensis*, *C. gigas*, and *C. virginica* (with a reliability of 100%). MgMDR2 was assigned with high support (99%) to a different cluster with other bivalve orthologues from *Brachiodontes pharaonic*, *A. farreri*, *P. maximus*, *C. virginica, C. gigas*, *C. angulata*, and an orthologue from the gastropod *L. gigantea*.

### 2.2. Gene Expression by RT-qPCR

#### 2.2.1. Expression of *mdr1* and *mdr2* in *M. galloprovincialis* Tissues

**Figure 5:**
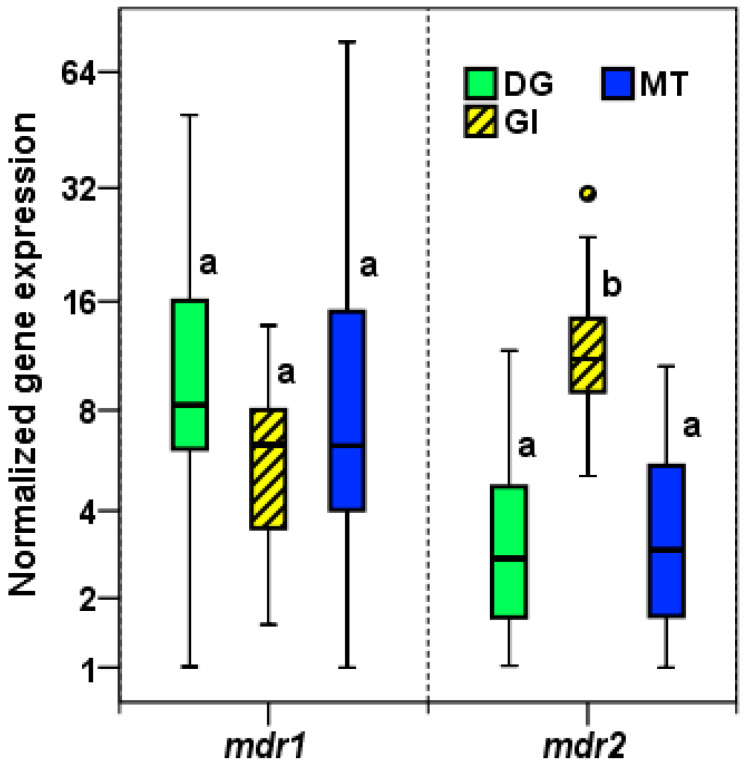
Normalized expression of *mdr1* and *mdr2* in digestive gland (DG), gill (GI), and mantle (MT) tissues as measured with qRT-PCR. The boxplots were obtained with SPSS 24.0. Boxes represent the lower and upper quartiles with medians. Bars represent the ranges for the data (*n* = 18). The circles represent the outliers (values between 1.5 and 3 interquartile ranges from the end of a box). For each gene, tissues not sharing the same letter are significantly different at *p* < 0.05 (Tukey’s HSD tests).

Four reference genes, *gapdh*, *cox1*, *rps2*, and *rps4*, previously selected in *M. galloprovincialis* [17], were used for normalization. The box and whisker plot graph (Figure 5) shows the normalized *mdr1* and *mdr2* expression in digestive gland, gill, and mantle tissues (*n* = 18, in each tissue). Statistical analyses with ANOVA and Tukey´s HSD test (Supplementary File S1) showed that *mdr1* gene presented a similar expression level in the three tissues, however *mdr2* gene expression was significantly higher in gill (*p <* 0.001) than in digestive gland and mantle tissues. Mean expression of *mdr2* in gill was 3.5 and 3.4 times higher than those in digestive gland and mantle, respectively.

#### 2.2.2. Expression of *mdr1*, *mdr2*, and *mrp2* in Presence of Okadaic Acid

The most stable reference genes were identified in each tissue using geNorm, NormFinder, and BestKeeper programs, and the results of the three algorithms were combined to obtain an overall rank (Supplementary Table S1). Pairwise variation was used to determine the optimal number of reference genes for normalization [31]. Therefore, three reference genes were selected in the digestive gland (*gapdh*, *rps4*, and *cox1*), gill (*rps4*, *cox1*, and *gapdh)*, and mantle (*rps4*, *gapdh* and *rps27*).

**Figure 6:**
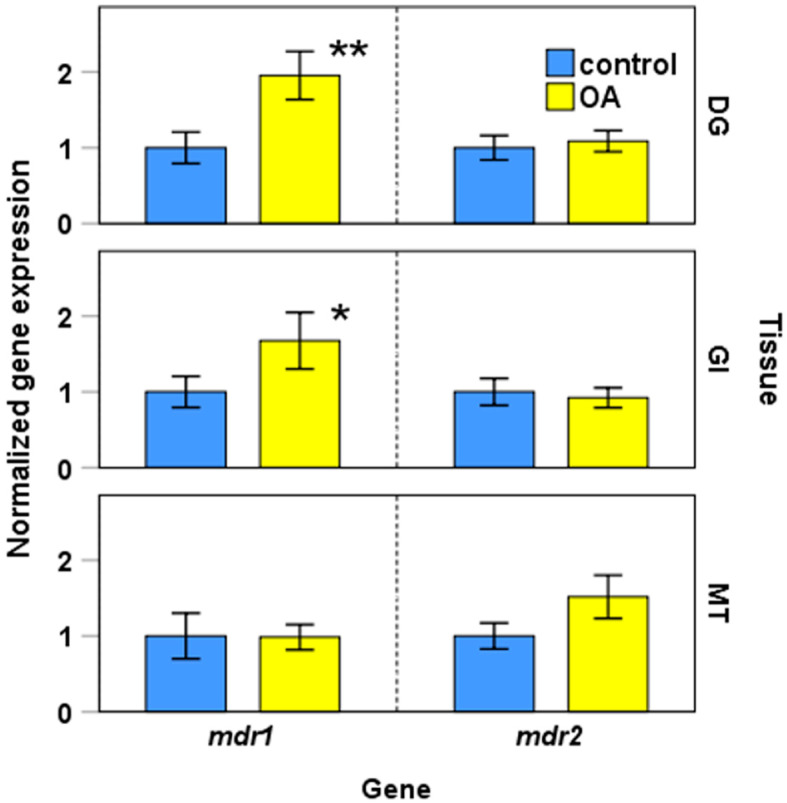
Normalized gene expression analysis in digestive gland (DG), gill (GI), and mantle (MT) tissues in the presence of okadaic acid (OA). Bars represent the mean ± standard error of the mean. Statistical analysis was performed with Student’s *t* test. * *p* < 0.05; ** *p* < 0.025.

The gene expressions of *mdr1* and *mdr2* in non-contaminated and okadaic acid-contaminated mussels are shown in Figure 6. Levels of okadaic acid in the digestive gland of control mussels were 1 ± 0.5 ng/g (mean ± SD) and in contaminated mussel 970 ± 380 ng/g. Statistical analyses showed significant differences in *mdr1* expression in digestive gland and gill tissues when control and okadaic acid groups were compared (Figure 6, Supplementary File S2). The presence of okadaic acid induced a 2.15-fold increase in the expression of *mdr1* in the digestive gland, and a 1.49-fold increase in the gill. No significant differences in the expression of *mdr2* were induced by the presence of okadaic acid.

**Figure 7:**
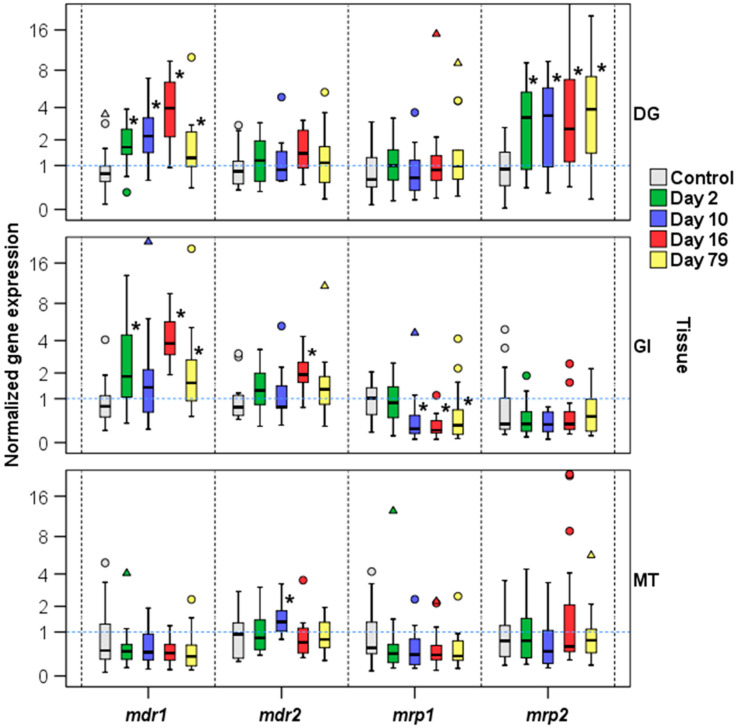
Normalized gene expression analysis of *M. galloprovincialis mdr1*, *mdr2*, *mrp1*, and *mrp2* during a cycle of accumulation-elimination of okadaic acid (OA) in the digestive gland (DG), gill (GI), and mantle (MT) tissues. Boxes represent the lower and upper quartiles with medians. The circles and triangles represent the outliers (values between 1.5 and 3 interquartile ranges from the end of a box). * indicates a significant expression against the control (* *p* < 0.05).

The normalized expression of *mdr* and *mrp* genes was also studied during a cycle (79 days) of accumulation-elimination of okadaic acid in mussels under a toxic tide of *Dinophysis acuminata* (Figure 7, Supplementary File S3).

**Figure 8:**
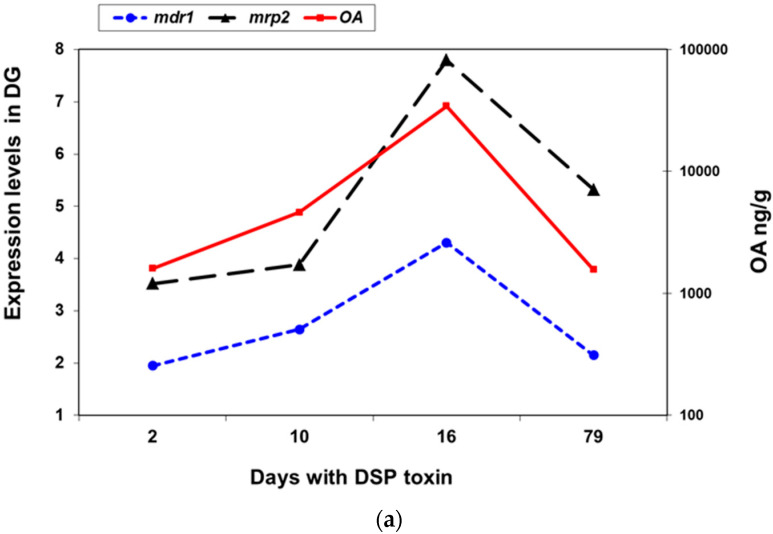

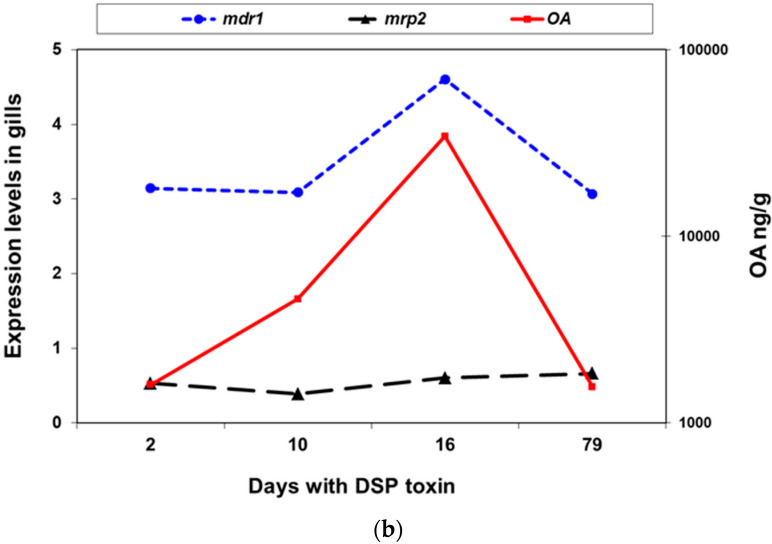
Gene expression analysis in the digestive gland (DG) (**a**) and gills (**b**) of *M. galloprovincialis mdr1* and *mrp2* during a cycle of accumulation-elimination of okadaic acid (OA). OA concentration in the DG is presented in ng/g wet weight.

Figure 8 shows the expression of the *mdr1* gene in the digestive gland and in the gills, respectively, and the evolution of the okadaic acid content in mussels. This figure also show the expression of *M. galloprovincialis mrp2* gene. As the okadaic acid content increases, *mdr1* expression is upregulated (a 4–5 fold increase) both in the gills and in the digestive gland. The evolution of the expression of the *mdr1* gene in these tissues follows a profile that is parallel to the content of okadaic acid in the mussel. The *mrp2* expression follows a similar pattern to *mdr1* expression in the digestive gland during the okadaic acid accumulation-elimination cycle. The expression of the *mdr2* gene remains stable without significant changes in both the gills and digestive gland. We have also found a correlation between OA concentration (log[OA]) and *mdr* 1 expression (log_2_*mdr1*) in the DG (*R* = 0.586; *p* < 0.01), showing that higher OA load induced higher mdr1 expression.

## 3. Discussion

In order to provide more information on the P-gp/MDR in bivalves, two full-length cDNA sequences of P-gp were successfully cloned from the mussel *Mytilus galloprovincialis*. The cDNAs were 4271 bp for *mdr1* and 4417 for *mdr2*. Open reading frames encoded 1307 and 1367 amino acid residues, respectively. The predicted molecular masses were 150.37 kDa for MDR1 and 143.91 kDa for MDR2. However, based on the sequon Asn-X-The/Ser, three *N*-glycosilation sequons were found in MDR1, while two were identified in MDR2, so the size should be higher than predicted. As demonstrated by Western blot, the size of *Perna viridis* P-gp is about 170 kDa [23], and a very similar size was found in *Crassostrea ariakensis* [22].

Analysis of the amino acid sequences of MDR1 and MDR2 predicted the structure organization of ABCB full transporters. The typical conserved structural domains of a eukaryotic ABC transmembrane transporter, such as the nucleotide binding domains NBDs, containing A-loop, Walker A, Q-loop, Walker B, D-loop, and H-loop were identified. The ABC transporter family signature (C motif), the one that specifically identifies the ABCB subfamily, was also present in both proteins (Figure 2 and Figure 3). These motives are essential for the functioning of P-gp in the transfer of energy to transport substrates across the membrane and to provide substrate specificity [32,33,34]. The deduced amino acid sequences and phylogenetic analysis of *M. galloprovincialis* MDR1 and MDR2 showed high homology with P-gp/MDR from bivalves and other organisms, supporting their identities as P-gp/MDR proteins.

It is interesting to note that after a homology search, MDR1 showed 95% identity with *Mytilus californianus* ABCB1, while MDR2 showed only 51% identity with both *M. galloprovincialis* MDR1 and ABCB1 of *M. californianus*. The phylogenetic analysis was consistent with this difference. MDR1 was assigned to a cluster with orthologues from other molluscs with a very high reliability, while MDR2 was assigned with high support to a different cluster with other bivalve orthologues.

The presence of two *mdr* genes encoding two different proteins is not specific for *M. galloprovincialis*. In fact, in humans, there are two distinct *mdr* genes that encode two P-gp proteins (MDR1 and MDR2/3), while in rodents, three *mdr* genes have been found, named Mdr1a, Mdr1b, and Mdr2. However, from the point of view of its functionality, only the *mdr1* gene in humans and *Mdr1a* and *Mdr1b* in rodents are considered to encode P-gp proteins. The products of these genes confer constitutive and inducible resistance against toxins. On the other hand, *mdr3* from humans and *mdr2* from rodents encode phospholipid translocating flippases [15,35]. Two Pg-gps have also been described in two fish species, *Pleuronectes americanus* and *Fundulus heteroclitus* [9].

Toxic tides associated with *Dinophysis* spp. often affect *M. galloprovincialis*. Manfrin et al. [4] studied the gene expression profile in this mussel induced by okadaic acid exposure using a specific DNA microarray with 7112 unique expressed sequences. The response observed in the digestive gland of OA contaminated mussels in a first phase showed an activation of putative defense mechanisms and/or physiological adjustments against the possible damage caused by the okadaic acid. Prado Alvarez et al. [36] studied the effect of okadaic acid on the clam *Ruditapes decussatus* in vitro but also in vivo using harmful algae bloom (HAB) simulation assay, in which clams were fed with cultures of *Prorocentrum lima*. These authors observed how OA and the simulated HAB caused damage to hemocyte functions and viability. Despite these facts, over long periods of exposure and in the presence of significant amounts of OA accumulated in their tissues, mussels remained alive and apparently healthy after exposure to *Dinophysis* or *Prorocentrun* toxic tides. This tolerance or insensitivity of bivalves to DSP toxins has been observed in many studies [37,38,39,40]. The study by Prado Alvarez et al. [36] cited above hardly reflects the concentrations of okadaic acid present in the environment or accumulated in the tissues of bivalves, in fact, in the HAB experiment, a maximum content of okadaic acid of 622.1 nanograms per gram was detected. In the experiments of Manfrin et al. [4], the food was supplemented only with 6.5 micrograms of okadaic acid every three days and the incorporated okadaic acid per gram of tissue was not indicated. On the other hand, Prego-Faraldo et al. [40] used concentrations of okadaic acid up to a maximum of 500 nM. Finally, Huang et al. [23] fed mussels, *Perna viridis*, with *Prorocentrum lima* and achieved levels of okadaic acid of around 25 ng per gram in gills.

There is abundant evidence supporting okadaic acid as a substrate of P-gp, as mentioned in the Introduction. Then, the interest in studying the expression of these two new characterized genes in *M. galloprovincialis* becomes obvious, and, for the aforementioned reasons, expression studies of the P-gp proteins in mussels under a toxic tide of *Dinophysis acuminata* were carried out.

At first, the expression of these genes was analyzed in three tissues where okadaic acid was accumulated. The lowest Cq (highest expression) corresponded to the *mdr2* gene in the gills (Figure 5). The normalized expression (Figure 6) showed that the *mdr1* gene presented a similar expression level in the three tissues. However, *mdr2* expression was significantly higher in gills, 3.5 and 3.4 times higher than in the digestive gland and mantle, respectively. It is known that gills take part in many biological functions [41], in fact, the gill–environment interface in bivalves is structurally comparable with blood–tissue barriers in vertebrate, such as the blood–brain barrier, which maintain homeostasis of sensitive tissues by controlling influx of nutrients and preventing entry of xenobiotics by ABC transporters [42].

*mdr* gene expression was also studied in contaminated mussels with an okadaic acid level of 970 ± 380 µg/g. Significant differences in both the digestive gland and gill tissues in *mdr1* expression were observed when control and okadaic acid groups were compared (Figure 7, File S2). The presence of okadaic acid increased 2.15 times that of the *mdr1* expression in the digestive gland and 1.49 times that of in gills. The role of the digestive gland as a major accumulator of DSP toxins was confirmed in a wide variety of studies in bivalves [43,44,45,46,47,48], and, furthermore, the importance of the gill–environment interface has been pointed out above.

Finally, the expression of *mdr* genes was monitored during a cycle (79 days) of accumulation-elimination of okadaic acid in mussels under a toxic tide of *D. acuminata*. Figure 8a,b show the expression levels of *mdr1* in the digestive gland and gills, respectively, and the evolution of the okadaic acid content. As in the previous experiment, the *mdr1* gene significantly increased its expression in the digestive gland and gills (4- to 5-fold) where okadaic acid accumulated in the mussels. When the bloom declines and okadaic acid falls to its initial levels, *mdr1* expression decreases in parallel both in gills and in the digestive gland. In the case of the *mdr2* gene, its expression remained fairly stable during the period studied, without statistically significant changes.

The magnitude of the observed changes in the *mdr1* expression falls within that which is expected. It has been reported that in fish, the upregulation of P-gp activity in response to xenobiotics is not high (1- to 2-fold) [49]. Miao et al. [34] found a similar increase in the scallop *Chlamys farreri* exposed to benzo (α) pyrene, a known inducer of phase I and II enzymes. These changes in expression are small compared to the change observed in the phase I enzyme CYP3A (5.3 to 12.7-fold) in the same experiment. Epel et al. [50] have suggested that the transporter activity level is already set to the expected historical load of xenobiotics, or that a small increase is adequate to protect the organism. In *M. galloprovincialis*, the 4- to 5-fold increase in *mdr1* gene expression together with the dramatic increase in accumulated okadaic acid levels, supports the idea of an important role played by the MDR1 protein in okadaic acid efflux out of cells in the digestive gland and gills. Furthermore, we have previously identified two multidrug associated proteins (MRP/ABCC) in *M. galloprovincialis*, and studied their expression in the presence of okadaic acid. A significant increase (6-fold) in the expression of MRP2 was observed in the digestive gland when the toxin was present [17]. The expression of these *mrp2* and *mdr1* genes together with the evolution of the okadaic acid content was also studied during the accumulation-elimination cycle of okadaic acid (Figure 8a,b). As in the case of *mdr1*, there was a significant induction (7–8 fold) of the expression of the *mrp2* gene in the digestive gland, as the content of okadaic acid increases in this tissue.

Broad and partially overlapping substrate and inhibitor specificities of P-gp and MRP-like transporters is a limiting factor in functional studies. In higher organisms, specific P-gp inhibitors are often used to check their role in the efflux of specific xenobiotics. Unfortunately, in bivalves, there are no data background that supports the specificity or even the functionality of these inhibitors. Huang et al. [23] used specific P-gp inhibitors, such as Verapamil and PGP-4008 in *Perna viridis* mussel fed with *Prorocentrum lima*. Surprisingly, they did not find significant differences in the accumulation of okadaic acid in the gills of this bivalve, although they did detect a decrease in MXR activity. However, a decrease in okadaic acid was detected when using the inhibitor cyclosporin A. Cyclosporin A, unlike Verapamil and PGP-4008, is a broad-spectrum MDR modulator that can prevent multiple ABC protein-mediated resistance with activity against P-gp, MRP, and the transporter BCRP. The BCRP protein was also recently identified and characterized in *M. galloprovincialis* in our lab (results not published). It appears to be a safety mechanism for organisms in which there are multiple transporters with partially overlapping substrate specificities [51]. Lin et al. [52] proposed that there is a compensatory mechanism in P-gP and MRP mediated resistance. The loss of one transporter can be functionally compensated by the over-expression of the other. Consequently, the expression of P-gp and MRP may constitute a functional defense network against xenobiotics.

A more effective way to approach functional studies with these transporters to evaluate the individual role of each one could be the use of micro RNAs (mi-RNAs). It is necessary to advance the knowledge of bivalve mi-RNAs and their interaction with ABC transporters. miRNA therapy that is related to ABC transporters has been identified as a promising strategy to radically treat metabolic diseases [53]. The design and use of mi-RNAs that target the MDR1 or MRP2 mRNA of *M. galloprovincialis* can be used to regulate the presence of these proteins and analyze their particular function in relation to okadaic acid.

Selective breeding programs with strains of mussels that have a lower toxin uptake and/or faster detoxification have been suggested to decrease the effects of increasingly frequent toxic episodes of microalgal origin that are threatening mussel production in Europe. Pino-Querido et al. [29] have estimated the heritabilities of okadaic acid concentration (as a balance of uptake and depuration) in 190 putative families of *M. galloprovincialis*. The variability between families and the estimates of heritability in that study prompted the launch of breeding programs to decrease the accumulation of toxins in cultured mussels during toxic tides of okadaic acid-producing microalgae. Thus, the study of the expression of the *mdr1* and *mrp2* genes in these families, as well as establishing their correlation with the molecular markers used in the previous hereditability study, could also offer insights into the specific role of MDR1 and MRP2 transporters in okadaic acid clearance in Mediterranean mussels. Furthermore, the knowledge of specific sequences of these genes allows the proposal of them as specific molecular markers in selective breeding programs.

## 4. Conclusions

The mussel *M. galloprovincialis* MgMDR1 and MgMDR2 were identified as P-gp/MDR/ABCB full transporters. Knowing its full sequence allows us to study its differential expression. We studied the expression of four genes in three different tissues during a long-term period (79 days), using a high number of samples. We also found a significant correlation between OA concentration and *mdr1* expression in the DG, showing that a higher OA load induced a higher *mdr1* expression. The expression profile of *mdr1*, together with the expression of *M. galloprovincialis mrp2*, a multidrug associated protein *(MRP/ABCC)*, allows the proposal that P-gp and MRP proteins are involved in the functional defense network against xenobiotics and in the resistance mechanisms to DSP toxins.

## 5. Materials and Methods

### 5.1. Cloning and Characterization of mdr Genes

#### 5.1.1. Animals and RNA Extraction

Adult mussels (*M. galloprovincialis*) were collected from culture rafts in the Rías of Ares-Betanzos and Muros-Noia (Galicia, NW, Spain). Mussels were dissected to separate the digestive gland, mantle, and gill tissues. The samples were treated with *RNAlater^®^* (Ambion, Applied Biosystems, Austin, TX, USA) according to the manufacturer’s instructions, and then they were stored at −20 °C prior to the RNA extraction.

Total RNA was extracted from ≈20 mg of digestive gland, gill, or mantle tissues. The *NucleoSpin^®^ RNA II* kit (Macherey-Nagel) for gill and mantle tissues, and the *RNAqueous*^®^ kit (Ambion, Applied Biosystems, Austin, TX, USA) for digestive gland tissue were used according to the manufacturer’s instructions. RNA was precipitated with 0.5 volume of lithium chloride (LiCl 7.5 M), in order to improve both RNA stability and the consecutive procedures of cDNA synthesis. The RNA pellet was dissolved in 50 μL of RNA Storage Solution (Ambion, Applied Biosystems, Austin, TX, USA) and treated with TURBO DNA-free™ (Ambion, Applied Biosystems, Austin, TX, USA). The integrity, quality, and quantity of RNA were determined using denaturating gel electrophoresis and a Nanodrop (ND-1000) spectrophotometer.

#### 5.1.2. Primer Selection

To confirm the presence of *mdr1* gene in *M. galloprovincialis*, an initial reaction was carried out using the primers designed by Franzelliti and Fabbri [30] for *M. edulis*. A fragment of expected size (381 bp) was obtained, and the other primers were designed starting with this first sequence. Moreover, using a similar *mdr* sequence in *M. galloprovincialis* (accession number ABO36618), new primers were designed to amplify the full-length cDNA of a new *mdr* gene that was called *mdr2*. All the primers used to obtain these sequences were synthesized by *Thermo* (Thermo Scientific Inc., Bremen, Germany), and are listed in Table 1.

#### 5.1.3. Cloning of cDNA Fragments and Rapid Amplification of cDNA Ends (RACE)

First-strand cDNA synthesis was performed using SuperScript^®^ III First-Strand Synthesis System for RT-PCR (Invitrogen, The Netherlands). First-strand cDNA was generated in a 20 µL final volume containing 2 µg total RNA, 2 µL of 10× RT buffer, 0.5 mM of each dNTP, 1 µL of 50 ng/µL random hexamers, 5 mM MgCl_2_, 2 µL of 0.1 M DTT, 1 µL (40 U) of RNaseOUT, 1 µL (200 U) of SuperScript III RT; the reaction continued for 10 min at 25 °C, 50 min at 50 °C, and 5 min at 85 °C. The cloning strategy to obtain *M. galloprovincialis mdr* genes is shown in Figure 1.

The PCR reactions were performed in a 50 µL final volume containing 2 µL cDNA template (1.5 µg), 0.2 mM of each dNTP, 0.2 µM of each primer, 5 µL of 10× HotMaster^TM^ Taq Buffer, 0.2 µL (1U) of HotMaster Taq DNA polymerase (Eppendorf, Hamburg, Germany). RT-PCR was performed in a Biometra thermal cycler with an initial denaturation at 94 °C for 3 min, followed by 35 cycles of denaturation (94 °C for 30 s), annealing (temperature varied depending on the primers, see Table 1) for 30 s, extension (72 °C for 1 min/kb), and a final extension step (72 °C for 2 min). The initial fragments of *mdr1* and *mdr2* were completed with the rapid amplification of the cDNA ends (5′- and 3′-RACE) using the SMART/SMARTer RACE cDNA Amplification kit (Clontech, Mountain View, CA, USA) following the manufacturer’s specifications. Some RT-PCRs were performed to reinforce the *mdr2* final sequence.

The single RT-PCR or RACE-PCR products of the expected size obtained for each gene were subcloned into a pGEM^®^-T Easy Vector (Promega, WI, USA). The clone screening was performed by PCR using M13 primers after DNA extraction with GenElute^TM^ Plasmid Miniprep kit (Sigma, St Louis, MO, USA). Plasmid DNA from positive clones was double-stranded sequenced using ABI Prism dRhodamine Terminator Cycle Sequencing kit (Applied Biosystems, Foster City, CA, USA).

#### 5.1.4. Sequence and Phylogenetic Analyses

Partial cDNA sequences were edited and assembled using BioEdit Sequence Alignment Editor version 7.0.5.3. Every sequence was represented by a minimum of five clones. Amino acid sequences were compared with protein sequences deposited in the GenBank, using the BLASTP algorithm [54] and FASTA [55]. A multiple alignment of selected sequences was constructed with Clustal Omega [56] using the available MDR amino acid sequences from bivalves and other organisms (default parameters, matrix = Blossum62, gap open = 10, and gap extension = 0.1). The two new sequences were deposited in the EMBL-EBI gene bank (accession numbers: FM999809 for *mdr1* and HF912273 for *mdr2*). The open reading frames of *mdr1* and *mdr2* were predicted through ORF Finder (Open Reading Frame Finder; National Center for Biotechnology Information (NCBI), Bethesda, MD, USA). Analyses of the amino acid sequences were conducted with Prosite (http://ca.expasy.org/prosite/ (accessed on 15 July 2020)) and Conserved Domain Database (CDD: NCBI, available from http://www.ncbi.nlm.nih.gov/Structure/cdd/cdd.shtml (accessed on 15 July 2020)). The Polyphobius algorithm http://phobius.sbc.su.se/poly.html (accessed on 20 July 2020) was used to predict the transmembrane topology and signal peptides from the amino acid sequences of *mdr1* and *mdr2*. PP Search software was used (http://www.ebi.ac.uk/Tools/ppsearch/ (accessed on 20 July 2020)) to locate *N*-glycosylation areas in proteins. Identity between sequences was calculated as the percentage identity (100 × number of matches/total number of amino acids). For the phylogenetic analysis of *mdr1* and *mdr2* amino acid sequences, the maximum likelihood method (MEGA X package) was used [57]. The trees were inferred using the JTT model of amino acid substitution and gamma distribution with five discrete categories. The statistical robustness of the nodes was evaluated by bootstrapping 2000 replicates.

### 5.2. Expression of mdr and mrp by RT-qPCR

#### 5.2.1. Animals, RNA Extraction, and cDNA Synthesis

To study the expression of *mdr1* and *mdr2*, two sets of *M. galloprovincialis* samples were collected from Ría de Arousa (Galicia, N.W: Spain): 18 control mussels and 15 mussels naturally exposed to okadaic acid (OA) producing *Dinophysis acuminata*. The animals were dissected to separate the digestive gland (DG), gill (GI), and mantle (MT) tissues. The comparative expression in the three tissues was analyzed in the 18 control mussels. To study the expression during a cycle of accumulation-elimination of OA, samples (five mussels from each different depth 1, 5, and 10 m) were collected at day 2, 10, 16, and 79 from the beginning of the bloom.

The samples were treated with RNAlater^®^ (Ambion, Applied Biosystems, Austin, TX, USA) according to the manufacturer’s instructions, and then they were stored at −20 °C prior to the RNA extraction. Total RNA was extracted as described above. First-strand cDNA synthesis was performed using iScript™ cDNA Synthesis kit (BioRad, CA, USA). First-strand cDNA was generated in a 20 µL final volume containing 0.6 µg total RNA, 4 µL of 5× iScript Reaction Mix, and 1 µL iScript Reverse Transcriptase; the reaction was allowed to continue for 5 min at 25 °C, 30 min at 42 °C, and 5 min at 85 °C.

#### 5.2.2. Primer Design and PCR Efficiency

Eight candidate reference genes (*gapdh*, *cox1 rps4 rps27*, *tif5a*, *act*, *nd4*, and *18S*) and two target genes (*mdr1* and *mdr2)* were used in the study of gene expression (Supplementary Table S1). The candidate reference genes were previously used for the normalization of RT-qPCR data in *M. galloprovincialis* by Lozano et al. [17].

Oligonucleotide primers were designed with the OligoAnalyzer 3.1 (http://eu.idtdna.com/analyzer/Applications/OligoAnalyzer/ (accessed on 30 July 2020)) from the sequences of Table 2. Oligonucleotides were synthesized by Thermo Scientific (Thermo Fisher Scientific, Inc., Waltham, MA, USA). The primer sequences and amplicon lengths are also listed in Table 2. The specificity of the primers was confirmed by the presence of a single peak in the melting curve, and by the presence of a single band of the expected size when PCR products were run in a 2% agarose gel. The identity of the amplicons was also confirmed by sequencing.

Various dilutions in triplets of a pool of all available cDNAs were used to generate the database for the determination of the PCR amplification efficiency (E) of each transcript [58]. Therefore, for each primer pair, a standard curve was obtained based on known quantities of cDNA, 5-fold serial dilutions corresponding to cDNA for *gapdh*, *cox1*, *rps27*, and *rps4* in digestive gland, gill, and mantle tissues. For *mdr1 and mdr2*, a 4-fold serial dilution of template cDNA was used in digestive gland, gills, and mantle. PCR efficiency (defined as percentage) was calculated with Bio-Rad iQ software V3.1 from the slope of the standard curve for each tissue. E = 10^−1/slope^ − 1 and E (%) = (10^−1/slope^ − 1) × 100. PCR efficiencies are listed in Table 2.

#### 5.2.3. Quantitative Real-Time RT-PCR and Data Analysis

Real time PCR was carried out using an iCycler iQ machine (BioRad, CA, USA). The RT-qPCR amplifications were performed in a 20 µL total volume containing 10 µL SsoFast EvaGreen Supermix (BioRad, CA, USA), 4 µL of 1:5 diluted cDNA (24 ng of cDNA), sense and antisense primers (400 nM each), and 4.4 µL PCR-grade water. As a control for genomic DNA contamination, an equivalent amount of total RNA without reverse transcription was tested for each gene. A negative control (without cDNA) was included in each assay. The cycle conditions were previously described in Lozano et al. [17]. A melting curve and a gel electrophoresis of each gene were performed in order to verify that a single PCR product was amplified for each set of primers. The threshold value was set manually to calculate the Cq values.

#### 5.2.4. Analysis of Gene Expression Stability

The data obtained were analyzed using three Microsoft Excel based software applications, geNorm V3.5 [59], NormFinder V0.953 [60], and BestKeeper V1 [58]. The Cq values were either used directly for stability calculations (BestKeeper analysis) or were first transformed to relative quantities (RQ) [61] using the gene-specific PCR amplification efficiency (geNorm and NormFinder analyses): RQ = (1 + E)^ΔCq^, where E is efficiency, Cq is gene expression level, and ΔCq = lowest Cq value of all samples of this gene.

#### 5.2.5. Gene Expression and Statistical Analysis

The Cq values were transformed to quantities (Q, non-normalized expression) by using the equation: Q = (1 + E)^−Cq^. The normalized gene expression was calculated as the ratio between Q and the normalization factor, the geometric mean of the quantities of the selected reference genes [17,62].

Statistical analyses were performed with an IBM SPSS Statistics 24.0 package. Data were tested for normality (Shapiro–Wilk test) and for homogeneity of variance (Levene’s test). Gene expression was log-transformed (base 2) to meet the requirements of normality and homogeneity of variances. Gene expression levels were compared using an analysis of variance (ANOVA), followed by Tukey’s HSD test to identify differences between the three tissues. The gene expression of *mdr1* and *mdr2* in non-contaminated (control group) and okadaic acid-contaminated (okadaic acid group) mussels was compared by a Student’s *t*-test. The results were considered significantly different when *p* < 0.05.

### 5.3. Toxin Extraction and Okadaic Acid Analysis

For the quantification of total okadaic acid (okadaic acid + conjugated forms), each digestive gland was dissected and the toxins were extracted by homogenization with 100% MeOH (1:4, *w*:*v*) with and Ultraturrax (IKA) at 15,000 rpm for 3 min, while maintained in ice. The homogenate was clarified by centrifugation at 48,000× *g* for 20 min. An aliquot of this extract was subjected to alkaline hydrolysis according to the Vale and Sampayo procedure which was slightly modified [63]. Briefly, the method consisted of adding 62.5 μL of 2.5 N NaOH to 0.5 mL of extract, heating it at 76 °C for 40 min in a closed vial, and finally neutralizing it by adding 62.5 μL of 2.5 N HCl. The analysis of the hydrolyzed extract after filtration through a 0.22 μm pore Nylon syringe filter (Membrane Solutions) was carried out by HPLC-MS/MS with an online SPE system made by a Jasco HPLC pump, a Rheodyne 6-way 2-position valve, and a Phenomenex Security Guard AJO-8367 (4 × 2 mm) as SPE column, according to the procedure of Regueiro et al. [64] which was slightly modified. The chromatographic separation was carried out with a Thermo Accela chromatographic system using a Phenomenex Gemini NX C18 50 × 2.1 mm, 3 µm column. The chromatographic gradient used NH_4_OH 6.7 mM as phase A, and MeCN 95% with NH_4_OH 6.7 mM as phase B, with a flow of 400 µL min^−1^. A mixture of 90 A:10 B was used as the loading phase for the SPE column. The chromatographic run started at 25% of phase B, which was maintained for 1.5 min, while the sample was loaded in the SPE column and the salts washed, then the contents of the SPE column were derived to the chromatographic column and a linear gradient, ending after 2.35 min at 95% B, was started. This proportion was maintained for 1.4 additional minutes, and, after that time, returned to the initial conditions to equilibrate the column for the next injection. The injection volume was 5 µL. The detection was made by to a Thermo Quantum Access MAX triple quadrupole mass spectrometer with a HESI-II electrospray interface, operated in negative ionization mode. The following conditions were used: spray voltage, 3000 V; sheath gas, 50 (nominal), auxiliary gas (5); vaporizer temperature, 110 °C; capillary temperature 360°, collision gas pressure, 1.5 mTorr; tube lens, 139. Two transitions, 803.5 > 255.1, and 803.5 > 563.4, with collision energies of 48 and 43, respectively, were used to quantify and confirm the identity of okadaic acid. The quantification was carried out by comparing the response obtained in the analysis of the samples with that of a reference solution of okadaic acid supplied by the NRC (National Research Council) of Canada.

## Figures and Tables

**Table 1:** Specific oligonucleotides for initial and RT-PCR or RACE amplification of the Mediterranean mussel *Mytilus galloprovincialis mdr1* and *mdr2*.

Primer Name	Sequence (5′-3′)	Target	Amplicon (bp)	Annealing T
*mdr1*				
MDR1F	CAGAGGTTCTATGACCCAGATGCAG	RT-PCR amplification	381	54.5 °C
MDR1R	GTTCTCACTCTCAGAGTCTAATGCAG			
MDR1F2	CCATTGCCAGAGCTTTGATYAGAGACCC	3′Race PCR	1067	65 °C
MDR1R2	CTGYCCTCCYGATAACTGAGCTCCAYGC	5′Race PCR	1830	65 °C
MDRMgFw	GTAGCAGCCCTGGTTATAGC	3′Race PCR	1463	64.9 °C
MDR End Nested3′	GTAGAAAGTGGTACACACCAGACTC	3′Race PCR	217	65 °C
*mdr2*				
MDR2 Start Rc3′F	TGCCAGAACCAGAATTATTGGGAT	RT-PCR amplification (+qRt Rv)	1290	50–56 °C
MDR2 Race3′ Fw 1	AAGAAGGGAGAAGAGGAAGAAAAGGA	3′Race PCR/RT-PCR amplif. (+Rv1&Rv2)	850/1161–1249	65 °C/55 °C
MDR2 Race3′ Fw 2	TCGTTATAACGCTCCTGAATGGCC	3′Race PCR	475	65 °C
MDR2 Race3′ Fw 3	TGGTTGGATGTATTGCTGCATGTTTGA	RT-PCR amplification + (Rv1&Rv2)	1070–1158	55 °C
MDR2 Race3′ Fw 4	GTGTTTGGAGCTATGGCTTTAGGACA	RT-PCR amplification (+Ultimo2 Rv)	1117	58 °C
MDR2 qRT Fw	GAGCCAAACTGGTAAGAGAGG	3′Race PCR	1082	65 °C
MDR2 qRT Rv	GTGGTGGAGCAACATTACCA	RT-PCR amplification (+Start Rc3′ F)		50–56 °C
MDR2 Fr Fw	TGGTGAGAGAGGAGCCCAGC	RT-PCR amplification	640	58 °C
MDR2 Fr Rv	AAATGCTGGCTGAACTCCAC			
MDR2 Rv1	TGCCAGTGTCTGTCCTGGATC	RT-PCR amplification (+Race3′ Fw 1& Fw 4)		55 °C
MDR2 Rv2	GGCAGGATCTGTGTTGCTTG	RT-PCR amplification (+Race3′ Fw 1& Fw 4)		55 °C
MDR2 End2 Rv	GCACTTACAATACACAGCAC	RT-PCR amplification (+Race Fw 4)		65 °C
MDR2Fin R3′F	AGCTGCTAGGAACGCTAACATTCATG	3′Race PCR	591	65 °C

**Table 2:** Primers used in this study for RT-qPCR, amplicon length (bp) for each primer pair and efficiency (E%) in each tissue (DG, digestive gland; GI, gill; MT, mantle).

Gene	Sense Primer (5′–3′)	Antisense Primer (5′–3′)	Amplicon Length	E% (DG)	E% (GI)	E% (MT)
*mdr1*	GTGGGCTCTAGCTCTTGTTG	GTCTTCCCAGCCTCCTCTAG	126	100.1	111.6	108.9
*mdr2*	TGGAGCCTATGCTCTTGGG	CAACATTACCAATGGACCACGC	131	99.8	105.1	91.7
*nd4*	CAGCCCCACCTAGTCTAAATC	AGCAAGCCCTAATAAAGCTCATC	114	105.8	100.5	100.9
*gapdh*	AGGAATGGCCTTCAGGGTAC	TCAGATGCTGCTTTAATGGCTG	114	99.3	107.3	96.5
*cox1*	TGCTCATTGGCATTGGGTGTC	AGTTCCTGCTCAGTCCATCTCAC	151	85.9	97.6	91.6
*rps27*	CGTGAATGTCCCAACGAAGAG	TGTTGCCTCTGGTTTGTTGA	114	92	101.2	97.3
*tif5a*	ACGCTACTTGACATTAACGATG	AGCTAGTTCTTCTCCCATAGC	171	96.9	99.3	104.6
*rps4*	TGGGTTATCGAGGGCGTAG	TCCCTTAGTTTGTGAGGACCTG	121	91.1	93.5	95.8
*act*	TCTTGATTTCGAGCAGGAAATG	GGATGGTTGGAATAATGATTCTG	138	91	111.3	100.3
*18S*	TCGATGGTACGTGATATGCC	CGTTTCTCATGCTCCCTCTC	84	99.2	87.7	94

## Data Availability

The data sequence presented in this study are openly available in EMBL-EBI gene bank (accession numbers: FM999809 for *mdr1* and HF912273 for *mdr2*) in the supplementary material.

## Supplementary Materials

The following are available online at https://www.mdpi.com/article/10.3390/toxins13090614/s1, Table S1: Rank of candidate reference genes in RT–qPCR, File S1: Statistical analysis of expression of *mdr1* and *mdr2* in *M. galloprovincialis* tissues, File S2: Statistical analyses of expression of *mdr1*, *mdr2*, *mrp1*, and *mrp2* in presence of okadaic acid, File S3: Statistical analyses of expression analysis of *M. galloprovincialis mdr1*, *mdr2*, *mrp1*, and *mrp2* during a cycle of accumulation-elimination of okadaic acid.
