# Supplementary material for: Expression Analyses of Genes Related to Multixenobiotic Resistance in Mytilus galloprovincialis after Exposure to Okadaic Acid-Producing Dinophysis acuminata
Source: Toxins (Basel). 2021 Sep 1;13(9):614. doi: 10.3390/toxins13090614 (PMC8471661; doi:10.3390/toxins13090614)
Supplement: Supplementary file 1 [file toxins-13-00614-s001.zip › Supplementary S1.pdf]

Supplementary Materials: Expression Analyses of Genes Related to Multixenobiotic Resistance in *Mytilus galloprovincialis* after Exposure to Okadaic Acid-Producing *Dinophysis acuminate*

Roi Martínez-Escauriaza, Vanessa Lozano, M. Luz Pérez-Parallé, Juan Blanco, José L. Sánchez and Antonio J. Pazos

Table S1. Rank of candidate reference genes in quantitative real-time reverse transcription–polymerase chain reaction (RT–qPCR), calculated by geNorm, NormFinder, and BestKeeper analysis.

Digestive gland (DG)									
**Rank**	**GeNorm**	**average M**	**Normfinder**	**Stability**	**BestKeeper**	**r**	**BestKeeper**	**SD**	**Overall**
**1**	*gapdh / rps4*	0.56	*gapdh*	0.088	gapdh	0.78	*gapdh*	0.76	***gapdh***
**2**	*gapdh / rps4*	0.56	*rps4*	0.105	rps4	0.77	*cox1*	0.84	***rps4***
**3**	*cox1*	0.9	*cox1*	0.150	tif5a	0.71	*rps4*	0.88	***cox1***
**4**	*act*	1.07	*tif5a*	0.180	act	0.67	*18S*	1.06	*tif5a*
**5**	*tif5a*	1.17	*rps27 / act*	0.188	rps27 / nd4	0.58	*rps27*	1.11	*act*
**6**	*rps27*	1.26	*rps27 / act*	0.188	rps27 / nd4	0.58	*act*	1.12	*rps27*
**7**	*18S*	1.41	*18S*	0.200	cox1	0.52	*tif5a*	1.19	*18s*
**8**	*nd4*	1.69	*nd4*	0.318	18S	0.38	*nd4*	2.15	*nd4*
**Gill (GI)**									
**Rank**	**GeNorm**	**average M**	**Normfinder**	**Stability**	**BestKeeper**	**r**	**BestKeeper**	**SD**	**Overall**
**1**	*gapdh / rps4*	0.63	*cox1*	0.088	*cox1 / rps4*	0.87	*cox1*	0.56	***rps4***
**2**	*gapdh / rps4*	0.63	*rps4*	0.092	*cox1 / rps4*	0.87	*rps4*	0.67	***cox1***
**3**	*cox1*	0.73	*gapdh*	0.093	*rps27*	0.86	*gapdh / 18S*	0.78	***gapdh***
**4**	*act*	0.82	*act*	0.106	*gapdh*	0.84	*gapdh / 18S*	0.78	*act*
**5**	*rps27*	0.88	*tif5a*	0.136	*act*	0.81	*rps27*	0.87	*rps27*
**6**	*tif5a*	1	*rps27*	0.137	*tif5a*	0.77	*act*	0.90	*tif5a*
**7**	*nd4*	1.08	*nd4*	0.139	*nd4*	0.65	*nd4*	0.92	*nd4*
**8**	*18S*	1.16	*18S*	0.188	*18S*	0.33	*tif5a*	1.08	*18S*
**Mantle (MT)**									
**Rank**	**GeNorm**	**average M**	**Normfinder**	**Stability**	**BestKeeper**	**r**	**BestKeeper**	**SD**	**Overall**
**1**	*gapdh / rps4*	0.8	*rps4*	0.094	rps4	0.76	*18S*	0.89	***rps4***
**2**	*gapdh / rps4*	0.8	*rps27*	0.114	tif5a	0.74	*rps4*	0.97	***gapdh***
**3**	*rps27*	1.06	*gapdh*	0.117	rps27	0.70	*gapdh*	1.15	***rps27***
**4**	*act*	1.2	*18S*	0.118	gapdh	0.69	*rps27*	1.18	*tif5a*
**5**	*tif5a*	1.34	*cox1*	0.126	act / 18S	0.68	*tif5a*	1.25	*18S*
**6**	*18S*	1.43	*act*	0.149	act / 18S	0.68	*cox1*	1.32	*act*
**7**	*cox1*	1.55	*tif5a*	0.172	cox1	0.65	*act*	1.42	*cox1*
**8**	*nd4*	1.89	*nd4*	0.339	nd4	0.49	*nd4*	2.37	*nd4*
